# The lymph node status as a prognostic factor in colon cancer: comparative population study of classifications using the logarithm of the ratio between metastatic and nonmetastatic nodes (LODDS) versus the pN-TNM classification and ganglion ratio systems

**DOI:** 10.1186/s12885-018-5048-4

**Published:** 2018-12-04

**Authors:** Carlos Fortea-Sanchis, David Martínez-Ramos, Javier Escrig-Sos

**Affiliations:** 1 Department of Surgery, Division of Colorectal Surgery, Consorcio Hospitalario Provincial de Castellón, Av. Doctor Clara, 19, 12002 Castellón, Spain; 2 Department of Surgery, Hospital General de Castellón, Av. Benicassim s/n, 12004 Castellón, Spain

**Keywords:** Colorrectal cancer, Lymph node, Prognosis, Log ratio

## Abstract

**Background:**

pN stage in the TNM classification has been the “gold standard” for lymph node staging of colorectal carcinomas, but this system recommends collecting at least 12 lymph nodes for the staging to be reliable. However, new prognostic staging systems have been devised, such as the ganglion quotients or lymph node ratios and natural logarithms of the lymph node odds methods. The aim of this study was to establish and validate the predictive and prognostic ability of the lymph node ratios and natural logarithms of the lymph node odds staging systems and to compare them to the pN nodal classification of the TNM system in a population sample of patients with colon cancer.

**Methods:**

A multicentric population study between January 2004 and December 2007. The inclusion criteria were that the patients were: diagnosed with colon cancer, undergoing surgery with curative intent, and had a complete anatomopathological report. We excluded patients with cancer of the rectum or caecal appendix with metastases at diagnosis. Survival analysis was performed using the Kaplan–Meier actuarial method and the Log-Rank test was implemented to estimate the differences between groups in terms of overall survival and disease-free survival. Multivariate survival analysis was performed using Cox regression.

**Results:**

We analysed 548 patients. For the overall survival, the lymph node ratios and natural logarithms of the lymph node odds curves were easier to discriminate because their separation was clearer and more balanced. For disease-free survival, the discrimination between the pN0 and pN1 groups was poor, but this phenomenon was adequately corrected for the lymph node ratios and natural logarithms of the lymph node odds curves which could be sufficiently discriminated to be able to estimate the survival prognosis.

**Conclusions:**

Lymph node ratios and natural logarithms of the lymph node odds techniques can more precisely differentiate risk subgroups from within the pN groups. Of the three methods tested in this study, the natural logarithms of the lymph node odds was the most accurate for staging non-metastatic colon cancer. Thus helping to more precisely adjust and individualise the indication for adjuvant treatments in these patients.

## Background

Colon cancer is the most frequent malignancy in both sexes in Western countries, with an incidence of approximately 471,000 cases per year and a mortality of 228,000 cases per year in Europe [1]. Lymph node involvement is the prognostic factor most directly related to survival and the disease-free interval. Thus, patients with stage I or II disease have a 5-year survival rate of more than 75% compared to 30–60% in patients at stage III or IV. In addition, the total number of lymph nodes analysed, both in cases at lymph node stage pN0 and in patients with tumour infiltration, has been shown to be a prognostic factor [2–4].

Until now, the pN stage in the TNM classification has been the “gold standard” for lymph node staging of colorectal carcinomas, but this system recommends collecting at least 12 lymph nodes for the staging to be reliable. However, new prognostic staging systems have been devised, such as the ganglion quotients or lymph node ratios (LNR) and natural logarithms of the lymph node odds (LODDS) methods, which aim to refine the construction of risk groups within the lymph node stage of this tumour and thus, provide better individualised oncological treatments for them.

The aim of this study was to establish and validate the predictive and prognostic ability of the LNR and LODDS staging systems and compare them to the pN nodal classification of the TNM system in a population sample of patients with colon cancer.

## Methods

### Patients

This was a multicentric population study which used data from the Valencian Community Tumour Registry—a registry of high-quality tumour samples which were included in the EUROCARE study [1]. The data used from this registry corresponded to the period between January 2004 and December 2007. The inclusion criteria were that the patients were: diagnosed with colon cancer, undergoing surgery with curative intent, had a complete anatomopathological report, and that the time and vital status at the last follow-up were clearly noted. We excluded patients with cancer of the rectum or caecal appendix with metastases at diagnosis, scheduled surgery with palliative intention without lymphadenectomy, scheduled surgery without resection, incomplete anatomopathological reports, a dubious vital status at the last control, and those with insufficient or no follow-ups noted.

### Variables

The study variables were: age, sex, tumour location, histology, degree of differentiation, and size, number of lymph nodes analysed, number of positive lymph nodes, TNM classification, condensed T and N stages, LNR, LODDS, adjuvant treatment, vital status and follow-up recurrence, follow-up time, overall survival, and disease-free survival.

Because all the data in the tumour registry is coded according to the 6th edition of the TNM, we had to adapt them to the guidelines for the 7th edition: category N was correctly readapted but the T category could not be adapted from the 6th to the 7th edition because the tumour registry contained insufficient data. As in other population studies, to minimise the effects of possible misclassifications, we used condensed TNM stages. Likewise, to reduce the number of subgroups, we organised the pN TNM classification category into 3 groups: pN0, pN1, and pN2. In terms of disease-free survival, the recurrence variable included patients who presented locoregional recurrence and those who presented distant metastases. The follow-up time we considered was from the date of surgery until the day of death, or the last day of follow-up in patients who did not die. This was because the tumour registry did not contain any clear definition of the date of diagnosis.

### LODDS and LNR

The LODDS values are defined as the log ([pLN + 0.5]/[nLN + 0.5]), where pLN corresponds to the number of positive lymph nodes and nLN to the number of negative ones. A value of 0.5 was added to both the numerator and the denominator to avoid a singularity error. The LNRs are defined as the percentage of positive lymph nodes from the total number of nodes analysed.

Cumulative summation of differences (CUSUM) graphs were used to distinguish differentiated groups within continuous-type prognostic variables (LNR and LODDS) following the method described by Barrio et al. [5], observing the changing trend in the curve in relation to another qualitative binary result variable (death or recurrence at follow-up). LNRs and LODDS were subsequently categorised for analysis (in their original continuous format) into three well-differentiated survival groups: the LNRs were grouped into 0 to 24%; 25 to 60%; and more than 60%, and the LODDS were grouped into values up to − 2; from − 2 to − 1; and greater than − 1.

### Statistical analysis

The variables were summarised by calculating the median and the interquartile range, or with frequencies and percentages. For the univariate analysis, the Chi-square test (Fisher test in small samples) was used to compare two qualitative samples, the Mann–Whitney U test was implemented to compare quantitative and dichotomous qualitative variables, and the Kruskal–Wallis test was utilised to compare quantitative variables to qualitative non-dichotomous ones. Survival analysis was performed using the Kaplan–Meier actuarial method and the Log-Rank test was implemented to estimate the differences between groups in terms of overall survival and disease-free survival. Multivariate survival analysis was performed using Cox regression. Values of *p* <  0.05 were accepted as the statistical significance cut-off level. Statistical analysis was carried out with the IBM-SPSS® program (version 22) for Windows and the CUSUM curves were calculated using the STATA® (version 12) program for Windows.

## Results

**Fig. 1:**
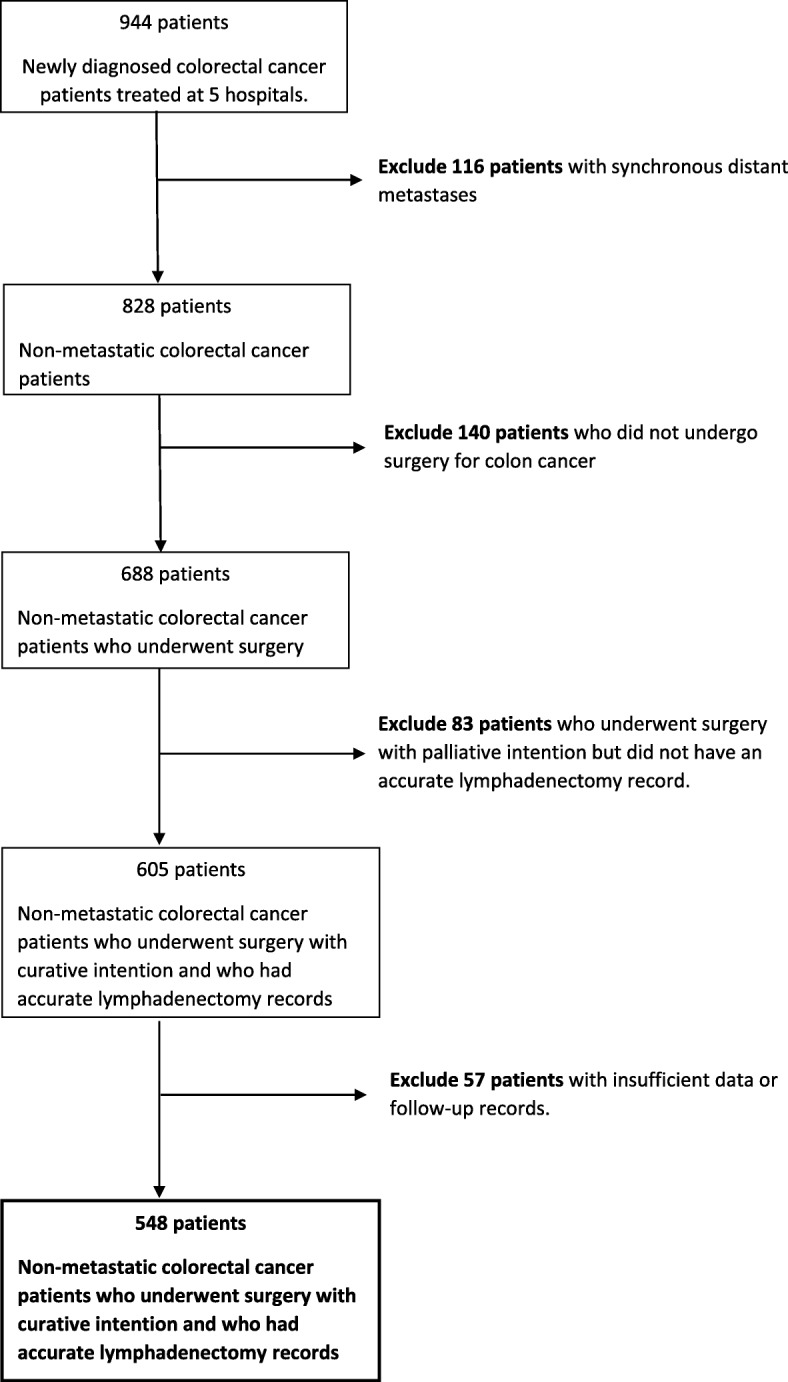
Enrolment of patients eligible for this study

During the 4 years of the study, 944 patients were diagnosed with colon cancer in our area, 396 patients were excluded from our work for different reasons, as shown in Fig. 1. Thus, 548 patients were finally analysed in this present study. The median follow-up period and range was 51 (30–64) months. The clinical and histopathological characteristics of the sample are shown in Table 1. The overall survival results and disease-free survival, according to the univariate and multivariate analyses, are shown in Table 2.

**Table 1:** Clinical and pathological data of the series

	LODDS1 (LODDS ≤ − 2)	LODDS2 (LODDS > − 2 to ≤ − 1)	LODDS3 (LODDS > − 1)	Total	*P*-value
n	349	187	12	548	
Age^a^	72 (62–79)	73 (64–79)	74 (56–81)	72 (63–80)	0.63
Grouped Age	75 (21.5%)	31 (16.6%)	3 (25%)	109 (19.9%)	
< 60	140 (40.1%)	79 (42.2%)	3 (25%)	222 (40.5%)	0.53
> 75	134 (38.4%)	77 (41.2%)	6 (50%)	217 (39.6%)	
Gender					
Female	151 (43.3%)	97 (51.9%)	4 (33.3%)	252 (46%)	0.11
Male	198 (56.7%)	90 (48.1%)	8 (66.7%)	296 (54%)	
Tumoral Location					
Right colon	128 (36.7%)	61 (32.6%)	6 (50%)	195 (35.6%)	0.73
Transverse colon	40 (11.5%)	17 (9.1%)	1 (8.3%)	58 (10.6%)	
Left colon	30 (8.6%)	14 (7.5%)	0 (0%)	44 (8%)	
Sigmoid colon	129 (37%)	84 (44.9%)	4 (33.3%)	217 (39.6%)	
Unknown	22 (6.3%)	11 (5.9%)	1 (8.3%)	34 (6.2%)	
Histology					
Adenocarcinoma	300 (86.5%)	151 (81.6%)	10 (83.3%)	461 (84.7%)	0.14
Mucinous	41 (11.8%)	32 (17.3%)	1 (8.3%)	74 (13.6%)	
Signet-ring cell	6 (1.7%)	2 (1.1%)	1 (8.3%)	9 (1.7%)	
Major Size (mm)^a^	45 (30–60)	40 (35–53)	40 (30–50)	45 (32–55)	0.49
Grade					
Unknown	9 (2.6%)	9 (4.8%)	1 (8.3%)	19 (3.5%)	0.025
I	98 (28.1%)	52 (27.8%)	1 (8.3%)	151 (27.6%)	
II	227 (65%)	108 (57.8%)	8 (66.7%)	343 (62.6%)	
III	15 (4.3%)	18 (9.6%)	2 (16.7%)	35 (6.4%)	
Adjuvant Chemotherapy					
No	262 (75.1%)	103 (55.1%)	6 (50%)	371 (67.7%)	< 0.001
Yes	87 (24.9%)	84 (44.9%)	6 (50%)	177 (32.3%)	
Number retrieved lymph nodes^a^	12 (8–16)	9 (5–13)	7 (5.5–11)	10 (7–15)	< 0.001
Cut-off retrieved lymph nodes					
< 12	178 (51%)	120 (64.2%)	10 (83.3%)	308 (56.2%)	0.002
≥ 12	171 (49%)	67 (35.8%)	2 (16.7%)	240 (43.8%)	
Number positive lymph nodes^a^	0 (0–0)	2 (1–4)	6.5 (4.5–10)	0 (0–1)	< 0.001
Lymph Node Ratio					< 0.001
0–24	349 (100%)	99 (52.9%)	0 (0%)	448 (81.8%)	
25–60	0 (0%)	80 (42.8%)	0 (0%)	80 (14.6%)	
> 60	0 (0%)	8 (4.3%)	12 (100%)	20 (3.6%)	
Condensed pT6					
T1–T2	84 (24.1%)	32 (17.1%)	2 (16.7%)	118 (21.5%)	0.16
T3–T4	265 (75.9%)	155 (82.9%)	10 (83.3%)	430 (78.5%)	
Condensed pN7					
N0	315 (90.3%)	31 (16.6%)	0 (0%)	346 (63.1%)	< 0.001
N1	34 (9.7%)	107 (57.2%)	2 (16.7%)	143 (26.1%)	
N2	0 (0%)	49 (26.2%)	10 (83.3%)	59 (10.8%)	
Condensed TNM stage					
I	81 (23.3%)	12 (6.4%)	0 (0%)	93 (17%)	< 0.001
II	234 (67%)	19 (10.2%)	0 (0%)	253 (46.2%)	
III	34 (9.7%)	156 (83.4%)	12 (100%)	202 (36.8%)	
Postoperative Death (90 days)					
No	324 (92.8%)	167 (89.3%)	12 (100%)	503 (91.8%)	0.27
Yes	25 (7.2%)	20 (10.7%)	0 (0%)	45 (8.2%)	
Follow-up General Mortality					
No	234 (67%)	97 (51.9%)	3 (25%)	334 (60.9%)	< 0.001
Yes	115 (33%)	90 (48.1%)	9 (75%)	214 (39.1%)	
Follow-up Recurrence					0.004
No	293 (84%)	139 (74.3%)	7 (58.3%)	439 (80.1%)	
Yes	56 (16%)	48 (25.7%)	5 (41.7%)	109 (19.9%)	
Follow-up Time (months)^a^	53 (38–66)	46 (17–63)	22 (15–49)	51 (30–64)	0.002

^a^Median (IQR: interquartile rang)

**Table 2:** General survival and disease-free survival: univariate and multivariate analysis

	General survival						Disease-free survival					
	Univariate			Multivariate			Univariate			Multivariate		
	HR	95% CI	*p*	HR	95% CI	*p*	HR	95% CI	*p*	HR	95% CI	*p*
Age	1.06	1.04–1.07	< 0.001	1.05	1.03–1.07	< 0.001	1	0.99–1.02	0.62			
Gender												
Male	1.08	0.76-1.52	0.67				1.01	0.69–1.46	0.99			
Female	1						1					
Location												
Right colon	1.95	0.94–4.03	0.186				1.07	0.45–2.57	0.41			
Transverse colon	1.74	0.78–3.90					0.81	0.28–2.34				
Left colon	1.12	0.46–2.74					0.37	0.09–1.49				
Sigmoid colon	1.89	0.82–3.89					1.13	0.48–2.66				
Unknown	1						1					
Histology												
Adenocarcinoma	0.43	0.19–0.98	0.12				0.30	0.11–0.81	0.30			
Mucinous Variant	0.41	0.17–0.99					0.28	0.09–0.85				
Signet-ring cell	1						1					
Grade												
Unknown	1.39	0.54–3.59	0.22				1.04	0.31–3.45	0.68			
Good	1.11	0.58–2.13					0.70	0.31–1.55				
Moderate	1.49	0.81–2.76					0.98	0.47–2.03				
Poor	1						1					
Major Size (mm)	1	0.99–1.01	0.96				1	0.99–1.01	0.399			
Number retrieved LN	0.97	0.95–0.99	0.007	1.07	0.99–1.15	0.045	0.99	0.97–1.02	0.99			
Less than 12	1.38	1.05–1.82	0.023				0.90	0.62–1.31	0.87			
12 or more	1						1					
Number positive LN	1.09	1.05–1.13	< 0.001	1.97	1.94–1.99	0.027	1.02	1.01–1.02	< 0.0001	1.08	1.01–1.15	0.02
Lymph Node Ratio (LNR)	1.01	1–1.02	< 0.001				1.02	1.01–1.03	< 0.0001			
0–24%	0.32	0.19–0.54	< 0.001				0.20	0.10–0.38	< 0.0001			
25–60%	0.60	0.34–1.09					0.46	0.22–0.97				
> 60%	1						1					
Condensed pT (6th Ed.)												
pT1–2	0.48	0.33–0.72	< 0.001	0.46	0.31–0.70	< 0.001	2.60	1.43–4.73	< 0.0001	2.27	1.24–4.15	0.008
pT3–4	1						1					
Condensed pN (7th Ed.)												
pN0	0.47	0.33–0.69	< 0.001				0.25	0.16–0.40	< 0.0001			
pN1	0.65	0.43–0.98					0.34	0.20–0.58				
pN2	1						1					
Condensed TNM stage												
I	0.31	0.19–0.52	< 0.001				0.21	0.10–0.47	< 0.0001			
II	0.78	0.59–1.03					0.63	0.42–0.93				
III	1						1					
LODDS	1.30	1.18–1.43	< 0.001	1.43	1.11–1.85	0.006	1.41	1.24–1.61	< 0.0001	1.21	1.01–1.46	0.036
LODDS1 (≤ −2)	0.28	0.14–0.55	< 0.001				0.20	0.08–0.51	0.005			
LODDS2 (> −2 to ≤1)	0.46	0.23–0.91					0.39	0.15–0.98				
LODDS3 (> 1)	1						1					
Adjuvant Chemotherapy												
Yes	0.63	0.46–0.85	0.003				1.93	1.33–2.81	< 0.0001			
No	1						1					

*LN* Lymph nodes. Multivariate study: variables in continuous format

**Fig. 2:**
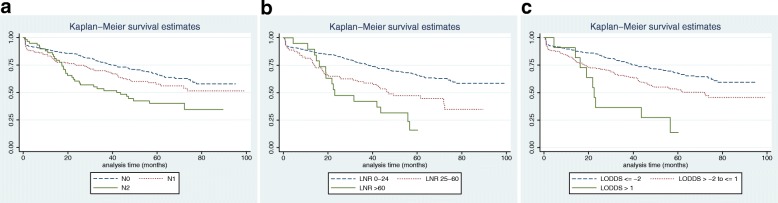
Overall survival according to the different staging methods: **a**. Condensed Category N (TNM 7th Ed.), **b**. LNR and **c**. LODDS

**Fig. 3:**
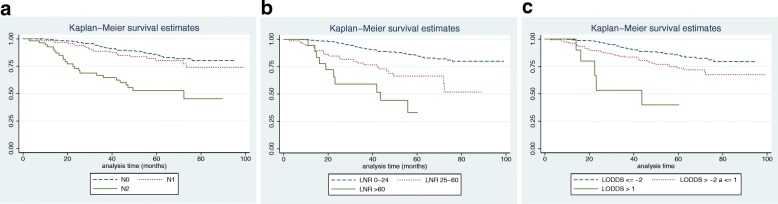
Disease-free survival according to the different staging methods: **a**. Condensed Category N (TNM 7th Ed.), **b**. LNR and **c**. LODDS

Overall survival and disease-free survival, comparing the pN classification with the LNR and LODDS, are shown in Figs. 2 and 3. For the overall survival (Fig. 2), groups with well-differentiated survival rates and with non-overlapping survival curves were observed in the three groups and there were significant differences (*p* <  0.0001) between all of them. However, the LNR and LODDS curves were easier to discriminate because their separation was clearer and more balanced. For disease-free survival (Fig. 3), the discrimination between the pN0 and pN1 groups was poor, but this phenomenon was adequately corrected for the LNR and LODDS curves which could be sufficiently discriminated to be able to estimate the survival prognosis.

**Fig. 4:**
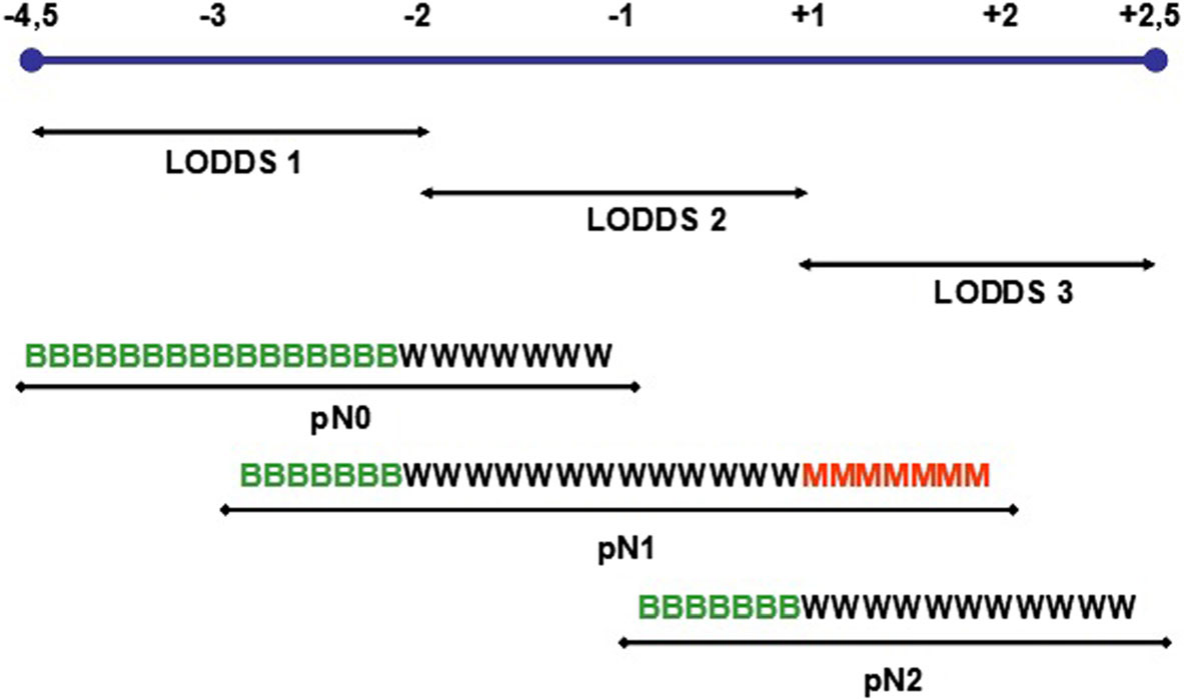
Correspondence between the LODDS, the condensed category N, and the prognosis

An analysis of the 3 pN groups, according to their corresponding LODDS and LNR results, lymph node analysis results, and overall survival is shown in Table 3. As shown, in the LODDS, the analysis of fewer lymph nodes was associated with a worse prognosis, and this relationship was the same for the number of positive lymph nodes. Similarly, the results shown in Table 3, and visually represented in Fig. 4 (where the prognosis was B, ‘better’, W, ‘worse’, or M, ‘much worse’) correspond to the different LODDS subgroups. In the pN0 group, LODDS differentiated groups with different prognoses while the LNR was unable to identify these differences. According to the different prognosis groups established by LODDS, within both the pN0 and pN1 groups there were prognosis subsets, and subsets with a very different prognosis were also distinguished in the pN2 group.

**Table 3:** Characteristics of the pN groups according to the LODDS results

	N0 LODDS 1	N0 LODDS 2	N1 LODDS 1	N1 LODDS 2	N1 LODDS 3	N2 LODDS 2	N2 LODDS 3
Lymph Nodes retrieved^a^	12	3	18	9	2	14	9
Positive Lymph Nodes^a^	0	0	1	2	2	6	8
Lymph Node Ratio^a^	0%	0%	7%	20%	100%	45%	84%
60 months Overall Survival	67%	57%	73%	53%	50%	46%	11%

^a^Median

## Discussion

LNR and LODDS analysis can distinguish risk subgroups with different survival rates more precisely than the condensed pN category alone. In the multivariate study, LODDS was identified as an independent risk factor for mortality and disease-free interval in non-metastatic colon cancer patients. After the determining presence of distant metastasis (M factor) and the condition of the primary tumour (T factor), the most important element in predicting the prognosis of colon cancer is lymph node involvement (N factor). Given its enormous importance, especially in terms of prognostic and therapeutic decisions, gaining a detailed picture of the lymph node status of patients with colon cancer should be a priority for clinicians involved in the diagnostic-therapeutic process of these patients.

Firstly, our results confirm that it is possible to create prognostic TNM-classification subgroups, based on the patient lymph node stage (pN), as shown in Figs. 2 and 3. Subgroups with well-differentiated survival rates can be identified using various classification systems. Nonetheless, in colon cancer, the total number of nodes analysed is a fully demonstrated prognostic factor [2–4]. In fact, this is the main drawback of the TNM classification because it only considers the number of affected lymph nodes and ignores the influence associated with the total number of lymph nodes analysed. Therefore, the new lymph node staging systems (LNR and LODDS) were created to try to provide a more detailed view of the lymph node status in patients by analysing both the total number of lymph nodes analysed and the total number affected.

Secondly, LNR analysis can identify subgroups with distinct prognoses in patients with non-metastatic colon cancer based on their lymph node staging, as widely reported in the international literature and as confirmed by the results we obtained in this work. This system has been shown to be superior to standard TNM classification, thus in 2010, Ceelen et al. performed a meta-analysis that included 16 studies with a total of 33,984 patients diagnosed with colon or rectal cancer and identified that the results of LNR analysis were an independent prognostic factor for stage III patients [6]. Furthermore, Moug et al. found evidence that the LNR results may be prognostically significant for every tumour stage [7]. Based on our results (Figs. 2 and 3) we can conclude that LNR analysis can more precisely pinpoint subgroups than the TNM classification, both in terms of overall and disease-free survival because the curves it produces are more differentiated from each other than those produced by the condensed N category. However, the main disadvantage of LNR-based grouping is that it cannot classify the prognosis of patients who do not have any affected lymph nodes. Thus, in the same way as the TNM classification for the pN0 group, every case with no positive lymph nodes is included in the same group (LNR 0%) regardless of the total number of lymph nodes analysed. This characteristic is very important for colon cancer because about 75% of patients submitted to surgical treatment do not have any affected lymph nodes when studied anatomopathologically [8].

Like the LNR curves, the LODDS analysis (Figs. 2 and 3) can also distinguish risk subgroups with different survival rates more precisely than the condensed pN category. The advantage of the LODDS-based grouping over LNR-based grouping is that it can differentiate risk subgroups within the N0 category as well as also differentiating risk groups within the pN1 and pN2 categories.

The LODDS lymph node classification system has been used in different types of cancer. It was first applied in breast and gastric cancers and is now being studied in colon cancer. In 2004, Vinh-Hung et al. were the first to use this novel statistical technique in a group of 83,686 patients with breast cancer [9]. In terms of prognostic discrimination, the results using this technique were better than those for the TNM classification and similar to the LNR grouping technique results among patients with affected lymph nodes. Thus, the LODDS method was considered superior because it can be used to study lymph node involvement in these patients at all classification levels. Following on from studies on breast cancer, Yildirim et al. questioned the usefulness of the LODDS method, because the results obtained from it were similar to those from the LNR technique, yet its mathematical calculation is more complicated [10]. However, after first using LODDS in breast cancer samples, Sun et al. subsequently also tested these techniques in a sample of 2547 gastric cancer cases and demonstrated the superiority of the LODDS technique over the TNM and LNR methods [11]. Nonetheless, several authors [12, 13] favour the LNR method with which they obtained results similar to those from the LODDS. Some authors, maintain that the results obtained with the LODDS are lower than expected and that this could reflect a false correlation between the N classification and the result [14]. Finally, Wang et al. designed a population study of 24,477 patients with stage III colon cancer in which they demonstrated the importance of the LODDS method [15]; they obtained better results both against the TNN and LNR classifications and so they considered it a better risk stratification technique. Chang et al. obtained similar results with a sample of 9644 patients with stage I–III cancer [16]. However, another study in a sample of 1297 patients with stage I–III cancer obtained better results with the LNR than with the LODDS [17]. In summary, the LODDS technique has multiple advantages over the other methods used for lymph node staging in various cancers, including in colon cancer [18, 19].

In our multivariate analysis of the multiple factors involved in the overall survival of colon cancer (Table 2), of those implicated in overall colon cancer survival, age was independently-weighted, the number of lymph nodes analysed was limiting, and the number of positive lymph nodes (pN) in pT3–T4 cases and the LODDS results themselves were all involved. Of note, when these other factors were present, the LNR method lost its status as an independent risk factor. Regarding the disease-free interval, only LODDS, the number of affected lymph nodes, and the advanced T category were identified as independent risk factors in our multivariate analysis. Chemotherapy is usually indicated in N+ patients or in T3 N0 patients with additional risk factors (such as obstruction or perforation). Thus, it is logical that in the multivariate study, chemotherapy is not a decisive factor for survival because its effect is already be encompassed within the pN.

Similar to our finding in this series, in the international scientific literature there is, on the one hand, a lot of evidence that both the T category of the TNM and the nodal stage are directly related to survival, as demonstrated in various studies [20–22]. On the other hand, the usefulness the LODDS and the LNR remains controversial. Several other studies agree with our results and also show a direct relationship between the results from LODDS and survival, without showing a similar relationship with the LNR technique. Thus, Arslan et al. demonstrated this relationship, only with the LODDS results, in a series of 444 patients with colon cancer [23]. Similarly, Persiani et al. used Cox regression to demonstrate the same relationship with the LODDS results, but not with those from LNR, in a series of 258 patients with non-metastatic colon cancer [24]. In a study including 17,632 cases, Huang et al. demonstrated that the LODDS method was more accurate than the LNR when calculating specific survival rates [25]. On the other hand, other authors have demonstrated that both methods are directly related to survival [9, 10]. But, in a study of 372 patients with gastric cancer, Liu et al. showed that the NRL technique was a better predictor of overall survival than the LODDS method [12]. Many of these discrepancies may be the result of statistical problems derived from the data collection itself and from the design and definition of the different variables.

According to our findings in this present work, the TNM classification should be complemented with the LODDS risk sub-group in clinical practice because the precision of this method has been demonstrated in the staging of risks. Thus, a patient with an increased risk according to LODDS will have a worse predicted survival rate than one only assessed according to the TNM classification. This fact becomes even more important in patients in which an insufficient number of lymph nodes could be obtained and analysed.

The main limitation of the work we present here is its retrospective population-study design. The great disadvantage of population studies is their inherent variability. This variability is derived both from differences in the study centres (5 centres in our case) and in the possible variation in the surgical techniques used, surgeons’ specialisations, and the anatomopathological sample analysis techniques used. In contrast, the advantage of population studies is that, by analysing all the patients in a specific area without any selection, they provide a global view of the real situation within a given period. This also implies that the results are representative only of the population studied, in our case the province of Castellon in Spain. Therefore, it is very important to compare the similarity of the characteristics of this study population (shown in Table 1) with those of other populations in order to assess the external validity of our results. As stated in the literature, a median of 10 lymph nodes are usually analysed, whereas different scientific societies recommend the analysis of at least 12 nodes. However, several articles, especially those such as ours in which a population analysis has been performed, were unable to reach this threshold number of lymph nodes [2, 8, 26]. Despite the analysis of a low number of lymph nodes, pN2 is not underestimated because at least 7 lymph nodes must be found to be positive in order to stage a patient as pN2. Nonetheless, the collection of fewer than 12 nodes can affect the confidence in the diagnosis of a true pN0 stage. Furthermore, obtaining only 10 lymph nodes does not dramatically affect this confidence level: the risk of misclassifying a pN0 after having analysed 10 lymph nodes is less than 2% [27].

Another limitation was our use of the T category from the 6th rather than the 7th edition of the TNM classification; this was because it was the version in effect during the period of data collection. However, to help reduce this bias, we allocated the patients either into a group with earlier tumours (stages T1 and T2) or into one with locally more advanced tumours (stages T3 and T4). Thus, minimising the effect of any modifications to the T factor classification implemented between these two editions of the TNM. In our province, the tumour registry for colon cancer was started in 2004 and here in this study we used the whole database dating back to its origin. However, because the validation of the cases we analysed was complex and we needed 5-year post-surgery follow-ups, the complete database could only be obtained up to 2012. The methods of diagnosis and anatomopathological analysis have not substantially changed in our area and so the results should be comparable to those more recently obtained. However, it would be interesting perform the comparison using a more up-to-date database.

## Conclusions

The pN classification is useful as a prognostic factor and remains the standard for lymph node staging because its measurement is very easy, and its groups have a clear and generally well-differentiated prognostic significance. However, the LNR and LODDS techniques can more precisely differentiate risk subgroups from within the pN groups.

Of the three methods tested in this study, the LODDS was the most accurate for staging non-metastatic colon cancer. This technique is slightly more difficult to calculate but, in turn, it is better able to distinguish patient prognoses than that derived from the TNM pN classification or the LNR method for N0 cases, especially in cases where an insufficient number of lymph nodes had been analysed. Therefore, the LODDS system complements the pN classification by improving the discrimination of the colon cancer prognosis in different groups, thus helping to more precisely adjust and individualise the indication for adjuvant treatments in these patients.

## Abbreviations

CUSUM: Cumulative summation of differences
LNR: Lymph node ratios
LODDS: Logarithms of the lymph node odds
